# Combination Relationship between Features of Person-Centered Care and Patient Safety Activities of Nurses Working in Small–Medium-Sized Hospitals: A Cross-Sectional Study

**DOI:** 10.3390/nursrep12040083

**Published:** 2022-11-15

**Authors:** Myoung Soo Kim, Young Ok Cho, Jiwon Park

**Affiliations:** 1 Department of Nursing, Pukyong National University, Busan 48513, Republic of Korea; 2 Hyosung City Hospital, Busan 48055, Republic of Korea

**Keywords:** social environment, nurse, patient safety, safety culture, self-concept

## Abstract

Perceived safety culture and nursing work environment are considered important prerequisites for a patient safety activity. Patient safety is also associated with person-centered care; however, few studies apply the person-centered care framework which includes staff attributes and care environment. This study aimed to examine the canonical correlations of person-centered care factors, including professional self-concept, patient safety culture, nursing work environment, and patient safety activities of nurses working in small–medium-sized hospitals. A cross-sectional survey was used. Participants included 171 nurses from seven small–medium-sized hospitals in Busan metropolitan city, in Korea. Data were analyzed using descriptive statistics, t-test, one-way analysis of variance (ANOVA), Pearson’s correlation coefficients, and canonical correlations. Two significant canonical variates were found. First, better professional self-concept, a positive patient safety culture, and better nursing work environment were associated with better patient safety care activities. Second, a negative patient safety culture and healthy nursing work environment were associated with a lack of communication between medical staff. Person-centered framework factors such as staff attributes and care environment were positively associated with patient safety activities. Based on the results, nurses in small–medium-sized hospitals should be highly aware of their professional self-concept. Moreover, nurses should be equipped with psychological safety and a healthy work environment to enhance patient safety activities.

## 1. Introduction

Patient safety is a top priority at healthcare facilities. Moreover, nurses are responsible for improving patient safety since they are frontline healthcare providers and comprise a large part of the healthcare workforce [1]. Patient safety activities include patient identification, medication confirmation, fall management, pressure ulcers, and hospital-acquired infections [2,3]. The existing literature highlights the factors influencing patient safety activities. Moreover, in recent times patient safety has been associated with person-centered care. Prior studies confirm an association between person-centered care, clinical effectiveness, better patient care processes [4], and health-related outcomes [5]. Furthermore, person-centeredness is not only a recurring theme in the recommendations for enhancing safety and best practices [6], but it has also been recognized by patients as an important determinant of safety. Therefore, person-centeredness has become a key concept in nursing and healthcare [7], and a theoretical model called the person-centered practice framework (PCPF) has been developed, which takes a whole-system approach to it [8]. The PCPF comprises four domains: prerequisites (i.e., nurse attributes), care environment (i.e., effective staff relationships and supportive organizational system), person-centered processes, and expected outcomes. According to the PCPF, to deliver person-centered processes which produce appropriate outcomes, prerequisites and the care environment must be adequately established [8].

Staff attributes are prerequisite factors for the delivery of person-centered care. This comprises professional competence, developed interpersonal skills, job commitment, ability to demonstrate clarity of beliefs and values, and self-knowledge [8]. Professional self-concept is the factor closest to staff attributes and is known to be associated with patient safety activity performance. Thus, a high degree of professional self-concept enhances patient safety activities [9]. According to a recent study, subcategories of professional self-concept were significant factors for medication-related quality care among geriatric hospital nurses [10]. 

The care environment includes multi-faceted characteristics, such as patient safety culture and nursing work environment, which affect patient safety activities [1,3,11]. Moreover, several studies have confirmed that the most common outcome variable of perceived safety culture was patient safety activities [12]. Given that innovative and supportive organizational cultures directly influence patient safety activities [13], a highly perceived patient safety culture can lead to recognizing and facilitating patient safety and preventing the omission of nursing tasks [14]. Therefore, a perceived safety culture is considered to be a strong antecedent of patient safety activities.

While perceived safety culture is a cognitive characteristic of the care environment, the nursing work environment refers to the physical and psycho-social environment [15]. Moreover, ‘staffing and resource adequacy’, ‘professional communication style’, and ‘nurse manager ability, leadership, and nurse support’ in the nursing work environment were associated with a higher level of patient safety [14,16]. In particular, poor nursing staffing was a predictor of more nursing care being left undone [17] or the omission of care [18], which was regarded as the factor influencing patient safety care activities [14,17]. Although the level of staffing is closely associated with patient safety and small–medium-sized hospitals in Korea experienced nursing shortages, there are no significant differences in patient safety activities between tertiary hospitals and small–medium-sized hospitals [19]. Hence, it is necessary to examine the relationship between organizational factors such as staffing and patient safety in an in-depth manner to enhance safety nursing activities in small–medium-sized hospitals.

While numerous studies demonstrated the effect of organizational and individual traits on patient safety activities, few studies have focused on the person-centered care framework. Moreover, prerequisite variables such as professional self-concept have not received enough attention. Further, the majority of prior studies have focused on tertiary or large-sized hospitals. Therefore, this study aimed to investigate the relationships of person-centered care variables and patient safety care activities of nurses working in small- and medium-sized hospitals.

**Hypothesis 1:**  *Person-centered care variables will exhibit combined relationships with patient safety activities.*

## 2. Materials and Methods

### 2.1. Study Design and Participants

A cross-sectional correlational study design was used. It is also a secondary analysis of the previous study [20] with the perspective of patient-centered care which has emerged as a key factor in determining patient safety. We employed non-probability sampling and selected seven small–medium-sized private hospitals in a metropolitan city of Korea; hospitals with 100–300 beds were invited to participate in a survey. The inclusion criteria for participating registered nurses (RNs) were: (a) those with more than six months of clinical experience in their current department and (b) those who directly perform patient care. Unit managers were excluded because they were more likely to respond in a positive way to the institution, potentially biasing the results. For each hospital, 20–30 questionnaires were provided. One of the researchers received permission to investigate from the nursing department in each hospital. One nurse employed by the research team at each hospital distributed questionnaires according to the guidance of the nursing department, and they collected the questionnaires as research assistants. When the respondents placed the completed questionnaire into the collecting box, research assistants collected the questionnaires. A total of 198 staff nurses were surveyed and 187 questionnaires were returned (response rate of 94.4%). After excluding 14 questionnaires that had missing answers, 171 questionnaires were included in the analysis. The required number of participants for the canonical correlation analysis was 10 participants per variable. Thus, 171 participants was judged to be sufficient for the analysis of nine variables.

The study adhered to the Strengthening the Reporting of Observational studies in Epidemiology (STROBE) guidelines for cross-sectional studies [21].

### 2.2. Instruments 

#### 2.2.1. Participant Characteristics

General characteristics comprised nine items: gender, age, marital status, job position, religion, shift type, educational level, working department, and total clinical experience. Patient safety-related characteristics comprised three items: number of hospital beds, patient safety-related education experience, and participation in the Accreditation Program for Healthcare Organization of Korea.

#### 2.2.2. Patient Safety Care Activities

Patient safety care activities were measured using a combination of a questionnaire developed by Park [3] that matched the six international patient safety goals and the healthcare accreditation items developed by the Korea Health Industry Development Institute [2]. The questionnaire comprised 24 items, which were classified into six subcategories: accurate patient identification (four items), communication between medical staff (five items), high-risk drug management (two items), accurate surgery/procedure confirmation (three items), infection prevention activities (seven items), and fall prevention activities (three items). Participants responded using a 5-point Likert scale, and higher scores indicated a higher level of patient safety care activities. In this study, the Cronbach’s alpha was 0.95.

#### 2.2.3. Professional Self-Concept

To measure professional self-concept, we used the Nurses’ Self-Concept Instrument (NSCI), which was developed by Angel et al. [22] and verified in Korean by Ryu and Kim [10]. It comprises 14 items, which were classified into the following four subcategories: knowledge of the nursing profession, care, relationship with members, and leadership. Items were rated on an 8-point Likert scale, ranging from 1 (strongly negative) to 8 (strongly positive); high scores indicated a positive professional self-concept. In this study, the Cronbach’s alpha was 0.93.

#### 2.2.4. Perceived Safety Culture

Perceived safety culture was measured using the Perceived Safety Culture Measurement developed by Lee [23]. It comprised 35 questions, in the following subcategories: leadership (nine items), teamwork (six items), patient safety knowledge/attitude (five items), patient safety policy/procedure (four items), and patient safety priority (three items). Items were rated on 5-point Likert scale; higher scores indicated a higher perception of patient safety culture. In this study, the Cronbach’s alpha was 0.89.

#### 2.2.5. Nursing Work Environment

To examine the nursing work environment, we used the Practice Environment Scale of Nursing Work Index, which was developed by Lake [24] and verified in Korean by Cho et al. [25]. It comprised 29 questions, which were classified into the following five subcategories: nurse participation in hospital affairs (nine items), nurse foundations for quality of care (nine items), nurse manager ability, leadership, and support of nurses (four items), staffing and resource adequacy (four items), and collegial nurse–physician relations (three items). Items were rated on a 4-point Likert scale, ranging from 1 = definitely disagree to 4 = definitely agree, and a higher score indicated a better perceived nursing work environment. In this study, the Cronbach’s alpha was 0.93.

### 2.3. Ethical Considerations

Prior to the study onset, we obtained ethical approval from the Institutional Review Board of University Pukyong National University (approval number: 1041386-201906-HR-32-02). The researchers then sent an official letter to the chief nursing officer of the target hospitals to obtain permission for data collection, in which they explained the purpose and method of the study. The letter also enclosed the study questionnaires. Participants were required to read the purpose of the study, the anonymity of participation, the possibility of withdrawal, the confidentiality of data, and the storage period. A small gift was provided to the participants as reward for their participation.

### 2.4. Data Analysis

Statistical analyses were conducted using IBM Statistical Package for the Social Sciences (SPSS) statistics 25.0 program. Descriptive statistics, t-test, and one-way analysis of variance (ANOVA) were used to evaluate participant characteristics as well as the distribution of study variables. Pearson’s correlation was conducted to examine correlations between variables. Canonical correlation analysis was performed after the assumption tests (i.e., normality, linearity, outliers, and multicollinearity). Canonical variates were interpreted as reliable if the structure coefficient was >0.40; thus, an explanatory power > 10% was considered a meaningful value. 

## 3. Results

### 3.1. Participants’ Characteristics

Of the participants, 162 (94.7%) were female, and the average age was 31.5 years old. The most common shift type was ‘3 shift’ (53.8%). Further, the majority (59.6%) of the sample held a Bachelor of Science in nursing or higher. Fifty-seven (33.3%) participants worked in the general ward. The average total clinical experience was 7.3 years, and 122 people (71.3%) had work experience of less than 10 years. Regarding hospital size, a majority of the participants (43.9%) worked at a hospital where the number of beds was less than 150. No significant difference in patient safety care activities were observed by general characteristics (Table 1).

### 3.2. Correlations between Study Variables

The mean scores of patient safety care activities, perceived safety culture, nursing work environment, and professional self-concept were 4.25 ± 0.57, 3.36 ± 0.35, 2.55 ± 0.41, and 5.84 ± 0.85, respectively. In terms of patient safety care activities, the highest mean score was observed in accurate patient identification (4.32 ± 0.67), whereas the lowest score was observed in communication between medical staff (3.87 ± 0.78). All subcategories of the patient safety care activities were significantly correlated with perceived patient safety culture (r = 0.27~0.36) and professional self-concept (r = 0.19~0.35). Regarding the nursing work environment, a very weak significant correlation or non-significant correlation with patient safety care activities (r = 0.04~0.27) was found (Table 2).

### 3.3. Canonical Correlations

Most assumptions for canonical correlations were met. There were two significant canonical variates. Wilk’s lambdas for the first and the second variates were 0.70 (F = 3.76, *p* < 0.001) and 0.86(F = 2.71, *p* = 0.003), respectively. The first canonical variate showed that a positive patient safety culture (0.89), better nursing work environments (0.41), and better professional self-concepts (0.73) were associated with better patient safety care activities including accurate patient identification (0.88), communication between medical staff (0.71), high-risk drug management (0.77), accurate surgery/procedure confirmation (0.76), infection prevention activity (0.85), and fall prevention activity (0.71). Furthermore, the second canonical variate revealed an association between negative perceived patient safety culture (−0.44) and poor nursing work environment (−0.75) and lack of communication between medical staff (−0.60) (Table 3).

## 4. Discussion

Prior studies show that hospitals with a large number of beds are likely to have a higher accident rate and a weaker correlation between patient safety initiatives and performances [26]. Hospitals with a large number of beds are perceived to possibly lack an excellent working environment and patient safety. However, the turnover rate of nurses in Korean hospitals with 100–300 beds is twice that of hospitals with 300 or more beds, which is expected to cause a major setback in patient safety activities due to the lack of basic nursing care [27]. Person-centered care variables showed combined relationships with patient safety activities in this study. These findings help to answer questions about the factors involved when trying to improve patient safety.

The mean score of the patient safety care activities was 4.28 points, which was similar to scores observed in recent studies [28,29]. However, it was slightly higher than scores of studies conducted prior to 2015 [30]. In Korea, the importance of patient safety has changed after the ‘Korean Patient Safety Act’, after which several patient safety strategies, such as voluntary patient safety reporting, have been implemented. The introduction of, and participation in, patient safety systems can enhance the level of patient safety activities in hospitals; therefore, this study reports an increase in the level of patient safety activities compared with prior studies. In particular, since patients can identify events such as medical errors and patient-centered communication, high-quality care provision must be dealt with from the patients’ perspective [6]. Meanwhile, hospitals have provided nurses with safety education through on-the-job or off-the-job training, which can influence the patient safety culture or working environment regardless of the experience for quadrennial healthcare accreditation. However, there was no difference in patient safety care activities according to the completion of safety education in this study. It seems likely that there might be intervening variables that link safety education and patient safety care activities. Therefore, it is crucial to analyze patient safety activities from a person-centered-care perspective.

The mean score of the professional self-concept was 5.81, which was similar to the scores found in studies targeting small–medium-sized hospitals [31]. However, it was slightly lower than that of long-term care hospitals, which was 6.02 points [10]. Nurses in long-term care hospitals are more likely to be supervising nursing assistants rather than performing direct care [32]; as they may perceive that they are doing important work, they may have a relatively high professional self-concept. Influencing factors for RNs’ professional self-concept were the self, role, and context [33]; the self is the RN who enacts the role in nursing practice, whilst the context refers to the clinical setting. Strong alignment between the self and context not only leads to a high professional self-concept but also enhances satisfaction with nursing role, staff retention, and improved quality of care [33]. Nurses working for small–medium-sized hospitals should perceive greater value in their role. 

The mean score of the perceived patient safety culture, which was regarded as having a direct effect on patient safety activities, was 3.36 points. This result is similar to that of previous studies [34,35]. However, it was arithmetically lower than that of the patient safety care activities using the same 5-point scale. Even though the importance and standards for patient safety strengthened, there is still a gap between the perceived patient safety culture and patient safety activities [29,36]. Perhaps culture change is a challenging and slow process, and develops over a long period of time [37]. Hence, changes in patient safety culture should be periodically reviewed and positive modification of safety culture should be induced. 

The level of the nursing work environment was relatively good, as a score higher than 2.5 points indicated a positive work environment [24]. However, the score was lower than that of nurses working in Magnet hospitals [38], indicating that the nursing work environment in this study had potential for improvement [14,28]. Therefore, it is necessary to continuously improve the nursing work environment with medical institution certifications. 

There are two significant canonical variates in this study. In the first canonical variate, professional self-concept—including staff attributes and better care environment including patient safety culture and nursing work environment—were associated with better patient safety activities. Although our study found a high correlation between professional self-concept and patient safety activities, it is difficult to draw conclusions as this study did not focus on professional self-concept and patient safety activities. However, nurses with a high professional self-concept were found to be in harmony with other medical professionals, as they were effective in performing their duties, and establishing the position of nurses in society [39]. Furthermore, powerlessness, isolation, and low self-esteem affected nurses’ perceived ability to ensure patient safety. Moreover, the perceived inability to perform in an autonomous manner influenced nurses’ professional self-concept [40]. 

In a recent study, a program for improving patient safety culture related to infection prevention among healthcare providers showed steady patient safety culture over time and decreased hospital-acquired infection [41]. Psychological safety has been an important issue in clinical settings due to the concern for patient safety, which is a key component of safety culture. A shared belief that team members will not be reprimanded, punished, or embarrassed for speaking up, sharing ideas, posing questions, raising concerns, or making mistakes, is critical for enhancing patient safety, including infection prevention [42] and drug management [43]. Contrary to developed Western countries, where the Patient Safety and Quality Improvement Act was established in 2005, healthcare institutions in Korea enacted the Patient Safety Act to improve the quality of care only after ten years. According to the reported data from Patient Safety Self-Report Learning System over five years from July 2016, mean patient safety reports were annually approximately 7500 cases; however, additional reports during the next one year reported approximately 10,000 cases. This indicated that the Act and the patient safety reporting system generated meaningful change in terms of positive psychological safety because the implementation of the patient safety reporting system emphasized a blame-free culture [44]. 

As it is widely known, work environment was also negatively associated with adverse events. A previous study showed that a better work environment reported a 45% decrease in adverse events such as wrong medication or dosage and a 32% decrease in falls [45]. Moreover, the interaction between work environment and professional self-concept affects accurate patient identification. Therefore, some factors in the person-centered frameworks, such as staff attributes and care environment, were positively associated with patient safety activities.

In the second canonical variate, negatively-perceived patient safety culture and nursing work environment were associated with a lack of communication between medical staff members. Patient safety culture and nurse—physician professional communication were positively associated with the perception of the quality of healthcare. In particular, safety culture deals with medical errors with non-punitive responses and facilitates effective nurse—physician communication [46]; thus, enabling mutual engagement can create a positive patient safety culture [47]. Nursing work environment contributes to better outcomes including collegial nurse–physician relations [48]. Similar results were observed in this study. Several studies found that work environment, including nursing shortages, work overload, and a lack of time, were also major barriers to person-centered care [49,50]. Patients experienced less teamwork between nurses and physicians through their own perceptions of inconsistency of communication between the staff, which in turn influenced consequences for patients as well as teamwork between nurse and physician [51].

### 4.1. Implications for Nursing Policy and Practice

In a clinical setting, it is unlikely that all four conditions related to person-centered nursing are always fulfilled. For example, an exponential increase in patients during the COVID-19 pandemic caused a disruption in the work environment (e.g., the number of beds and medical staff), person-centered processes, and outcomes. In such a situation, what kind of efforts can ensure patient safety? First, reinforcing professional self-concept and psychological safety is one way to improve the quality of activities associated with patient safety. Empowering nurses could improve the overall patient safety culture [52] because nurses with enhanced professional self-concept feel psychologically safe, which means that they deal properly with emotional distress, and tend to voice their concerns about safety issues. They are also better able to access the resources, information, and support that are required to perform their work in harsh environments. Second, to improve prerequisites and work environment, building communication channels for patient safety activities is necessary. One study in the recent literature suggests that artificial intelligence technology can substitute expensive human-controlled facilitators to achieve better communication training [53]. Therefore, strategies enhancing prerequisites and care environment should be developed for improving patient safety.

### 4.2. Limitations

This study examined the relationship between staff attributes, care environment, and patient safety care activities using a person-centered perspective. However, it has several limitations. First, as this study employed a non-probable sampling method, a sampling bias may be likely. Therefore, caution should be administered while generalizing the results of this study. Second, potential discrepancies between actual and perceived patient safety care activities cannot be overruled because this study did not employ objective measures. Therefore, future studies should use the probable sampling method and observations of actual patient safety care activities to rectify these limitations.

## 5. Conclusions

This study aimed to explore the combination relationship between features of person-centered care and patent safety activities of nurses working in small–medium-sized hospitals. The first variate showed that person-centered care variables were positively associated with all patient safety activities. Moreover, in the second canonical variate, perceived care environments were positively related with communication between medical staff. Our findings suggest that high professionalism, a good working environment, and a higher-perceived patient safety culture positively correlated with nurses performing patient safety activities. Since variables regarding person-centered care had a combination relationship with patient safety activities, it is pivotal to have comprehensive perspectives to increase nurses’ patient safety activities rather than focusing on sole aspects of staff attributes or the care environment. Additionally, the low-perceived patient safety culture, in particular, was related to the lack of communication between medical staff. Since poor communication can exacerbate various negative outcomes (e.g., medical errors, patient harm, patient dissatisfaction), standard person-centered education on patient safety is necessary for all staff in the hospital to improve overall patient safety. In this regard, nurses and organizations need to raise awareness of person-centered care and discuss together the individual efforts that are required for nurses to provide patient safety activities and what measures can be prepared for nurses and patients in the organizational aspect.

## Figures and Tables

**Table 1:** Patient Safety Activities according to Characteristics (N = 171).

Characteristics	Categories	n (%)	Patient Safety Activities	
			M ± SD	t/F (*p*)
Gender	Female	162 (94.7)	4.28 ± 0.54	−0.59 (0.557)
	Male	9 (5.3)	4.18 ± 0.63	
Age (years) (M ± SD = 31.5 ± 6.8)	<30	32 (18.7)	4.31 ± 0.67	1.46 (0.228)
	30–39	50 (29.2)	4.15 ± 0.56	
	40–49	66 (38.6)	4.35 ± 0.47	
	≥50	23 (13.5)	4.32 ± 0.51	
Marital status	Married	51 (29.8)	4.23 ± 0.61	−0.73 (0.468)
	Unmarried	120 (70.2)	4.30 ± 0.52	
Job position	Staff nurse	146 (85.4)	4.27 ± 0.53	−0.73 (0.469)
	Charge nurse	25 (14.6)	4.35 ± 0.64	
Religion	Christian	22 (12.9)	4.29 ± 0.54	0.16 (0.924)
	Catholic	6 (3.5)	4.14 ± 0.43	
	Buddhism	35 (20.5)	4.29 ± 0.54	
	Non-religion	105 (61.3)	4.31 ± 0.52	
	Others	3 (1.8)	3.46 ± 0.52	
Shift	Two shift	15 (8.8)	4.33 ± 0.62	0.13 (0.882)
	Three shift	92 (53.8)	4.26 ± 0.57	
	Fixed	64 (37.4)	4.29 ± 0.50	
Educational status	College	69 (40.4)	4.33 ± 0.51	0.93 (0.354)
	Above BSN	102 (59.6)	4.25 ± 0.57	
Working unit	General ward	57 (33.3)	4.17 ± 0.59	1.38 (0.243)
	Nursing care service ward	45 (26.3)	4.35 ± 0.50	
	Special unit	36 (21.1)	4.24 ± 0.56	
	Operating room	33 (19.3)	4.41 ± 0.50	
Total experience in nursing (years) (M ± SD = 7.3 ± 5.0)	<3	37 (21.6)	4.31 ± 0.59	0.39 (0.757)
	3~<5	26 (15.2)	4.25 ± 0.66	
	5~<10	59 (34.5)	4.23 ± 0.50	
	≥10	49 (28.7)	4.33 ± 0.50	
Numbers of bed	<150	75 (43.9)	4.21 ± 0.54	1.25 (0.288)
	150~<200	50 (29.2)	4.31 ± 0.56	
	200~<300	46 (26.9)	4.36 ± 0.54	
Experience of safety education	Yes	119 (69.6)	4.30 ± 0.56	
	No	52 (30.4)	4.25 ± 0.52	0.54 (0.589)
Experience of healthcare accreditation	Yes	69 (40.4)	4.34 ± 0.57	
	No	102 (59.6)	4.24 ± 0.52	1.22 (0.225)

BSN = Bachelor of Science in Nursing; M = mean; SD = standard deviation.

**Table 2:** Correlation between Perceived patient safety culture, Safety, Nursing Work Environment, Professional self-concept and Patient Safety Care Activities (N = 171).

Variable	M ± SD				Patient Safety Care Activities					
		1	2	3	4	5	6	7	8	9
		r(*p*)								
1. Perceived patient safety culture	3.36 ± 0.35	1.00	0.67 (<0.001)	0.45 (<0.001)	0.36 (<0.001)	0.35 (<0.001)	0.27 (<0.001)	0.30 (<0.001)	0.29 (<0.001)	0.28 (<0.001)
2. Nursing work environment	2.55 ± 0.41		1.00	0.37 (<0.001)	0.13 (0.083)	0.27 (<0.001)	0.04 (<0.001)	0.11 (0.131)	0.13 (0.076)	0.14 (0.058)
3. Professional self-concept	5.84 ± 0.85			1.00	0.23 (0.002)	0.19 (0.008)	0.23 (0.002)	0.22 (0.003)	0.35 (<0.001)	0.23 (0.002)
4. Accurate patient identification	4.32 ± 0.67				1.00	0.58 (<0.001)	0.67 (<0.001)	0.62 (<0.001)	0.55 (<0.001)	0.46 (<0.001)
5. Communication between medical staff	3.87 ± 0.78					1.00	0.48 (<0.001)	0.51 (<0.001)	0.45 (<0.001)	0.39 (<0.001)
6. High-risk drug management	4.43 ± 0.71						1.00	0.72 (<0.001)	0.65 (<0.001)	0.51 (<0.001)
7. Accurate surgery/procedure confirmation	4.41 ± 0.62							1.00	0.77 (<0.001)	0.60 (<0.001)
8. Infection prevention activity	4.41 ± 0.60								1.00	0.72 (<0.001)
9. Fall prevention activity	4.23 ± 0.77									1.00

**Table 3:** Predictors of Patient Safety Care Activities of nurses working in small and medium-sized hospitals (N = 171).

	Canonical Variates	
	1	2
Set 1. Organizational/Individual factor set		
Perceived patient safety culture	0.89	−0.44
Nursing work environment	0.41	−0.75
Professional self-concept	0.73	0.32
Set 2. Patient Safety Care Activities		
Accurate patient identification	0.88	−0.05
Communication between medical staff	0.71	-0.60
High-risk drug management	0.77	0.29
Accurate surgery/procedure confirmation	0.76	0.02
Infection prevention activity	0.85	0.28
Fall prevention activity	0.71	−0.04
Total redundancy set 1	9.63	2.41
Total redundancy set 2	11.73	0.72
Canonical correlation	0.44	0.29
Significance test F(*p*)	3.76 (<0.001)	2.71 (0.003)
Variance explained	0.19	0.08

## Data Availability

The datasets generated and analyzed during the current study are not publicly available but are available from the corresponding author upon reasonable request.
